# Neurotransmitters as Modulators of Neural Progenitor Cell Proliferation During Mammalian Neocortex Development

**DOI:** 10.3389/fcell.2020.00391

**Published:** 2020-05-26

**Authors:** Lei Xing, Wieland B. Huttner

**Affiliations:** Max Planck Institute of Molecular Cell Biology and Genetics, Dresden, Germany

**Keywords:** neocortex, neurotransmitter, neural progenitor cell, cell proliferation, development

## Abstract

Neural progenitor cells (NPCs) play a central role during the development and evolution of the mammalian neocortex. Precise temporal and spatial control of NPC proliferation by a concert of cell-intrinsic and cell-extrinsic factors is essential for the correct formation and proper function of the neocortex. In this review, we focus on the regulation of NPC proliferation by neurotransmitters, which act as a group of cell-extrinsic factors during mammalian neocortex development. We first summarize, from both *in vivo* and *in vitro* studies, our current knowledge on how γ-aminobutyric acid (GABA), glutamate and serotonin modulate NPC proliferation in the developing neocortex and the potential involvements of different receptors in the underlying mechanisms. Another focus of this review is to discuss future perspectives using conditionally gene-modified mice and human brain organoids as model systems to further our understanding on the contribution of neurotransmitters to the development of a normal neocortex, as well as how dysregulated neurotransmitter signaling leads to developmental and psychiatric disorders.

## Introduction

During mammalian brain development, the formation of the central nervous system (CNS) results from a series of events, which begins with the neural induction and the proliferation of the NPCs (Goodman and Shatz, 1993). In the early developing neocortex, neuroepithelial cells (NECs) function as the primary NPCs and undergo symmetric proliferative divisions to expand the neocortical NPC pool (Götz and Huttner, 2005; Lui et al., 2011; Florio and Huttner, 2014). At the onset of neurogenesis, NECs transform into apical (or ventricular) radial glia (aRG), which undergo mitosis at the ventricular surface and reside in the ventricular zone (VZ) of the developing neocortex (Götz and Huttner, 2005; Rakic, 2009; Lui et al., 2011; Florio and Huttner, 2014; Wilsch-Bräuninger et al., 2016). In virtually all mammals, aRG are thought to possess high proliferative capacity to both amplify themselves and give rise to basal progenitors (BPs), including basal intermediate progenitors (bIPs) and basal (or outer) radial glia (bRG) (Götz and Huttner, 2005; Rakic, 2009; Fietz et al., 2010; Hansen et al., 2010; Lui et al., 2011; Reillo et al., 2011; Florio and Huttner, 2014; Wilsch-Bräuninger et al., 2016). BPs delaminate from the ventricular surface and migrate to the subventricular zone (SVZ), where they typically reside and undergo mitosis to give rise to cortical neurons, which are destined for six different cortical layers (Götz and Huttner, 2005; Rakic, 2009; Lui et al., 2011; Florio and Huttner, 2014; Wilsch-Bräuninger et al., 2016). At later stages of development, either following neurogenesis or concomitant with still ongoing neuron production, NPCs switch their fate to generate glial cells, such as astrocytes and oligodendrocytes (Lee et al., 2000). In order to guarantee the proper construction of the complex neocortex, each step in this developmental sequence must be under precise spatial and temporal regulation. While significant progress has been made in understanding how NPC–intrinsic factors contribute to a balanced NPC proliferation, there are still open questions about the regulation of NPC proliferation by environmental cues, such as neurotransmitters.

Among several categories of cell-extrinsic signals, neurotransmitters have gained attention as important factors to influence CNS development (Cameron et al., 1998; Nguyen et al., 2001; Ojeda and Avila, 2019), although the classic role of neurotransmitters is in neuronal communication by acting as synaptic chemical messengers in the mature CNS. Indeed, neurotransmitters mediate developmental processes such as cell proliferation (Haydar et al., 2000), neuronal differentiation (Salazar et al., 2008), neuronal migration (Komuro and Rakic, 1993; Murthy et al., 2014), synaptic maturation (Fu et al., 2012), neurite growth (Anelli et al., 2013) and cell death (Ikonomidou et al., 2001). For example, serotonin controls the migration of caudal ganglionic eminence-derived interneurons into the neocortex (Murthy et al., 2014). The GABA receptors along developing inhibitory axons sense GABA release and promote presynaptic maturation to shape the pattern of synapse formation and distribution (Fu et al., 2012). Glutamate induces neuronal apoptosis, which is mediated via activation of calpain and caspase-3 proteases as well as the translocation of apoptosis inducing factor (Zhang and Bhavnani, 2006). Several recent studies strongly suggest that neurotransmitters could act as growth regulators or morphogen-like signaling molecules to regulate NPC proliferation during cortical development (Represa and Ben-Ari, 2005; Côté et al., 2007). In this review, we summarize our current knowledge on the regulation of neocortical NPC proliferation by different neurotransmitters during mammalian brain development and discuss future research perspectives in studying the involvement of neurotransmitters in neocortical development under both physiological and pathological conditions. We do not discuss in detail the synthesis and metabolism of any individual neurotransmitter, nor their role in other developmental processes beside progenitor proliferation, as these aspects have been intensively reviewed previously (Cameron et al., 1998; Nguyen et al., 2001; Represa and Ben-Ari, 2005; Ojeda and Avila, 2019).

## NPC Proliferation Regulated by Neurotransmitters

### GABA

During mammalian brain development, GABA, the main inhibitory neurotransmitter in the mature brain, excites cortical cells due to the high expression level of the Na^+^-K^+^-2Cl^–^ cotransporter (NKCC1) (Hübner et al., 2001) and low expression level of K^+^-Cl^–^ transporter member five (KCC2) (Owens and Kriegstein, 2002; Lee et al., 2005). As one of the most abundant neurotransmitters detected in the developing brain, GABA appears in the germinal zones, intermediate zone and layer I of the cortical plate during early stages of development (Haydar et al., 2000). Starting as early as E9.5 in mice, the GABAergic neurons generated from subcortical structures are gradually migrating into the developing neocortex, and these neurons could serve as the source of releasable GABA in the neocortical wall (Tanaka and Nakajima, 2012).

Although GABA is the most studied neurotransmitter in the context of regulating the proliferation of NPCs, there is apparent controversy about the trophic effect of GABA during neocortical development. Upon binding of GABA to GABA_*A*_ receptors, which in cultured E16–E19 rat neocortical tissue explants have been shown to be expressed in the VZ NPCs (presumably in aRG), Cl^–^ ions diffuse through these ion channels along their concentration gradient (LoTurco et al., 1995). The NPCs in the VZ of developing rat neocortex thus lose intracellular Cl^–^, which leads to membrane depolarization and the increase of intracellular Ca^2+^ concentration through the activation of voltage-gated calcium channels (VGCCs) (LoTurco et al., 1995). This increase of intracellular Ca^2+^ concentration, induced by GABA, is potentially involved in the inhibition of DNA synthesis of VZ NPCs and decreases their proliferation rate in the cultured tissue explants of developing rat neocortex (LoTurco et al., 1995). The same study also reported that the effects of GABA in inhibiting DNA synthesis in VZ NPCs can be blocked by modulating the Cl^–^ concentration using a GABA_*A*_ receptor antagonist (LoTurco et al., 1995). In line with this, another study (Andang et al., 2008) suggested that GABA inhibits cell cycle progression and therefore decreases proliferation of mouse embryonic stem cells and neural crest stem cells, which express glutamic acid decarboxylase (GAD) and functional GABA_*A*_ receptors. The underlying mechanisms include phosphorylation of the critical factor in the S/G2 DNA-damage checkpoint complex, histone H2AX, by phosphatidylinositol-3-OH-kinase-related kinase (PIKK) upon membrane hyperpolarization following GABA_*A*_ receptor activation (Andang et al., 2008). It has recently been shown that mouse VZ NPCs become more hyperpolarized at later developmental stages and that experimental membrane hyperpolarization shifts the transcriptional program and division mode of VZ NPCs to a later developmental stage, in which VZ NPCs generate two daughter IPs instead of amplifying themselves (Vitali et al., 2018).

However, it has also been reported that GABA_*A*_ receptor activation stimulates cell proliferation and renewal in a culture system of isolated NPCs from developing mouse brain. The increased proliferation rate was found to be due to an up-regulation of ciliary neurotrophic factor (CNTF) receptor expression, which in turn enhanced the trophic effect of CNTF (Fukui et al., 2008b). A follow-up study from the same research group further showed that GABA_*B*_ receptor activation led to a significant increase in the capacity of isolated mouse cortical NPCs in forming neurospheres, which has been supported by the analyses of GABA_*B*_R1-null mice (Fukui et al., 2008a). Thus, in the isolated mouse NPC culture system, GABA seems to be able to increase the proliferation of these progenitors through two separate mechanisms involving the recruitment of different types of GABA receptors and different growth-stimulating factors. The synthesis and release of growth factors and neuropeptides from NPCs in the developing neocortex can indeed be stimulated by neurotransmitters and may play a role in regulating NPC proliferation together with neurotransmitters (Fukui et al., 2008b; Yuzwa et al., 2016).

Regarding the contradicting findings between these studies, obvious explanations beside species differences would be the micro-environmental difference between tissue explant culture and isolated NPC culture, where different NPC populations are being studied, as well as the difference in developmental stage. Nevertheless, all these *in vitro* studies suggest that there is a direct effect of GABA in regulating NPC proliferation, with the direction of the effect being species-, region- and environment-dependent. However, surprisingly, gene-modified mice which have only 0.02% of GABA circulating in the embryonic brain due to the knockdown of the GABA-synthesizing enzymes GAD65 and GAD67 did not show altered brain histogenesis, including cortical layering (Ji et al., 1999). A possible explanation of the lack of adverse phenotypes could be that other neurotransmitter systems compensate for the malfunction induced by the loss of GABA, including modulation of cortical NPC proliferation and migration, possibly by glutamate and glycine, both of which are able to depolarize NPCs in the germinal zones of developing rodent neocortex (LoTurco et al., 1995; Flint et al., 1998). In addition, a more rigorous evaluation of cellular morphology and ultrastructure, cell density as well as the cellular composition of the developing neocortex is needed to further uncover developmental defects of these GAD-knockdown mice.

**FIGURE 1:**
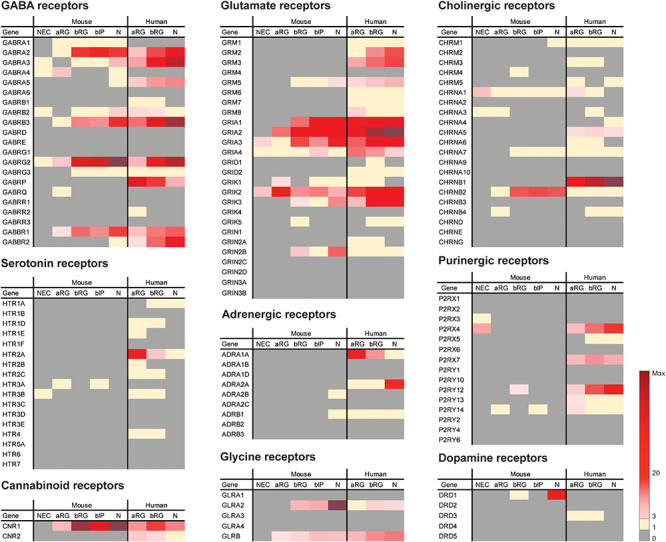
Previously published sets of transcriptomic data (Florio et al., 2015; Albert et al., 2017) were analyzed here for the mRNA expression levels of neurotransmitter receptors in embryonic mouse and fetal human neocortex. FPKM values of neurotransmitter receptors in the indicated isolated cell populations of embryonic mouse (E9.5 for NEC, E14.5 for aRG, bRG, bIP and N) and fetal human (12–13 wpc) neocortex are indicated by the color scale shown at bottom right. Note that N (neuronal fraction) in fetal human neocortex includes bRG in the G1 phase of the cell cycle. Key observations from the analyses can be summarized as follows. (1) Four neurotransmitter receptors, GRIA3, GRIK2, CHRNA1, and P2RX4, were found to be expressed in mouse NECs, however, at low levels (FPKM = 3.9, 5.1, 6.6, 5.5, respectively). This suggests that the involvement of neurotransmitter signaling in NEC expansion during mouse cortical development is presumably limited. (2) Of the neurotransmitter receptors that are expressed in both embryonic mouse and fetal human neocortex, the majority showed the highest expression levels in the N fraction, such as GABRA2, GABRB3, GABRG2, GRIA2, GRIK3, and GLRA2. These receptors are most likely expressed on the cell surface of neurons where they may receive the respective neurotransmitter signal. (3) All human neurotransmitter receptor-encoding genes presented in the figure have orthologs in mouse. Thus, an expression observed in one species but not the other indicates a differential expression pattern of the neurotransmitter receptor between cortical cells in mouse and those in human. For example, GABRA5, GABBR2, GRM2, GRM3, CHRNB1, and ADRA2A are potentially involved in neuronal functions only in human, but not mouse, during neocortical development. In contrast, GRIN2B, CHRNB2, and DRD1 are potentially involved in neuronal functions only in mouse, but not human, during neocortical development. (4) Of the neurotransmitter receptors only expressed in fetal human but not embryonic mouse neocortex, GABRP, HTR2A, ADRA1A, P2RX7, and CNR2 showed a greater expression in aRG and/or bRG than in N, which raises the possibility that the activation of these receptors could be of relevance for NPC proliferation during the development and even the evolutionary expansion of the human neocortex.

The alterations in proliferation of neocortical NPCs induced by the external application of GABA *in vitro* demonstrated that GABA has the potential to directly regulate NPC proliferation, a conclusion consistent with the finding that the opposite effects are observed upon blocking GABA receptors (LoTurco et al., 1995). This suggests that endogenously synthesized GABA in the developing neocortex regulates neurogenesis in rodent germinal zones, including both VZ and SVZ. Interestingly, the effects of GABA on NPC proliferation are completely opposite in the VZ NPCs (aRG) versus SVZ NPCs (BPs) of developing mouse neocortex, potentially due to activation of different receptor subtypes (Figure 1) and triggering different signaling mechanisms (Haydar et al., 2000). For example, the most highly expressed GABA receptor subunits in the mouse NPC populations are GABRA2 and GABRG2, both of which showed a relatively higher expression level in BPs (bRG and bIP) compared to APs (aRG). Thus, depending on the *in vitro* experimental conditions or the *in vivo* environment that the NPCs reside in, GABA signaling may exhibit different impacts on the proliferation of NPCs in developing neocortex.

### Glutamate

Glutamate is the main excitatory neurotransmitter in the mature CNS. Through binding to different types of receptors, glutamate is essential for maintaining various cognitive functions including learning and memory (Riedel et al., 2003; Mattson, 2008). Glutamate receptors can be categorized into two main classes: (1) ionotropic glutamate receptors (iGluR), which include three types of receptors: N-methyl-D-aspartate (NMDA) receptors, α-amino-3-hydroxy-5-methylisoxazole-4-propionic acid (AMPA) receptors, and kainic acid (KA) receptors; and (2) metabotropic glutamate receptors 1–8 (mGluR1–8) (Riedel et al., 2003).

During development, glutamate is detectable in the germinal zones of developing mouse neocortex as early as E12, potentially released by the Cajal-Retzius cells in the marginal zone (del Rio et al., 1995; Haydar et al., 2000). Among iGluRs, AMPA/KA receptors are the first ones to appear and are highly expressed by NPCs in the germinal zones of the embryonic rodent and fetal human neocortex (Figure 1) (LoTurco et al., 1995; Haydar et al., 2000; Maric et al., 2000). Through activating AMPA/KA receptors, glutamate decreases DNA synthesis of the NPCs in the germinal zones, and hence their proliferation, in rat organotypic slice cultures (LoTurco et al., 1995; Haydar et al., 2000).

The NMDA receptor is also involved in regulating NPC proliferation in developing mouse neocortex, albeit indirectly. Calcium imaging in cultured mouse neocortical slices suggested that MAP2–positive cortical neurons, but not nestin–positive NPCs in the VZ, are responsive to an NMDA antagonist (Hirasawa et al., 2003). Through regulating the expression levels of components of the Notch pathway and increasing the synthesis of brain-derived neurotrophic factor (BDNF), chronic exposure to the NMDA antagonist caused sustained proliferation of NPCs in the VZ (Hirasawa et al., 2003). In line with the finding that NMDA receptor activation inhibits cortical NPC proliferation in the developing mouse neocortex, NPCs isolated from developing rat neocortex, which are believed to transiently express NMDA receptor subunits, also showed a decreased proliferation when exposed to an NMDA receptor agonist (Yoneyama et al., 2008).

In contrast, elongated GFAP–positive NPCs, presumably radial glial cells, that express NMDA receptor subunits, dissociated from fetal human neocortex, responded to glutamate and an NMDA antagonist in a completely opposite manner compared to mouse NPCs. Glutamate significantly enhanced the proliferation rate of isolated human NPCs *in vitro*, and the increased proliferation could be inhibited by a specific NMDA receptor antagonist (Suzuki et al., 2006). The same study also showed that AMPA receptors, KA receptors and mGluRs are most likely not involved in the proliferation of radial glial cells induced by glutamate (Suzuki et al., 2006).

Among the mGluRs, it has been reported that mGluR5 is involved in the modulation of NPC proliferation in developing rat and human neocortex, where this receptor is expressed (Figure 1) (Boer et al., 2010; Zhao et al., 2011, 2012). In human, mGluR5 activation stimulates both ERK and JNK pathways, which leads to promotion of NPC proliferation. The human NPCs with activated mGluR5 also showed an increased level of cyclin D1, which results in cell cycle progression underlying the increased proliferation of NPCs (Zhao et al., 2011). In mouse, blocking mGluR5 function by a selective mGluR5 antagonist reduced proliferation and increased cell death of mouse forebrain NPCs during development, while the activation of mGluR5 increased the number of proliferating NPCs (Di Giorgi-Gerevini et al., 2005). In line with this, NPCs of mGluR5 knockout mice also exhibited decreased proliferation compared to those of wildtype mice, both *in vitro* and *in vivo* (Di Giorgi-Gerevini et al., 2005).

### Serotonin

Serotonin has been postulated to exert a role in cortical development, as cortical serotonin arises from placental sources at the onset of neurogenesis and from embryonic serotonergic afferents at later developmental stages in both mouse and human (Bonnin et al., 2011). Even though both the endogenous serotonin system in the embryonic hindbrain and placenta can be sources to supply the embryonic forebrain with sufficient serotonin starting from E10.5 and throughout the development of the mouse brain (Bonnin et al., 2011), there are no serotonin receptors, of any subtype, expressed at significant levels in the germinal zones of the developing mouse neocortex (Figure 1) to receive and amplify the readily available serotonin signals (Bonnin et al., 2011; Fietz et al., 2012; Florio et al., 2015). This has also been suggested by data from early *in vitro* studies using a rat organotypic slice culture system, which have shown no effect of serotonin on cortical NPC proliferation as the number of BrdU-labeled cells were similar between serotonin-treated and untreated rat neocortex slices (Dooley et al., 1997).

However, *in vivo* studies aiming to understand the effects of serotonin on cortical development using transgenic mouse models with altered serotonin levels in the embryonic neocortex have suggested that the proliferation rate of cortical progenitors is decreased by serotonin (Côté et al., 2007; Cheng et al., 2010). A double knockout mouse model for the serotonin-degrading enzymes, monoamine oxidase A (MAOA) and monoamine oxidase B (MAOB), exhibited significant reductions in Sox2–positive cells and Tbr2–positive bIPs in the SVZ at E17.5 and P2, but not at earlier developmental stages (Cheng et al., 2010). Although MAO metabolizes both serotonin and dopamine, it was suggested that the decrease in NPC abundance in MAO knockout mice was indeed caused by the increased level of serotonin, not dopamine (Cheng et al., 2010). In contrast, however, a knockout mouse model for the serotonin-synthesizing enzyme tryptophan hydroxylase 1 (TPH1) also showed a decreased number of BrdU-positive cortical progenitors in the VZ (Côté et al., 2007), which leaves the role of serotonin in NPC proliferation unclear.

Compared to the contribution of serotonin and its receptors to neuronal migration and maturation, for which there are several studies, very little is known about the effects of serotonin on the proliferation of cortical NPCs. Recent comparative transcriptomic studies have revealed a differential expression pattern of serotonin receptor 2A (HTR2A) in cortical NPCs between mouse and human (Florio et al., 2015; Mayer et al., 2019), which may point to a potential role of serotonin and HTR2A in regulating proliferation of human NPCs. However, no effects on progenitor proliferation have been observed when treating cultured human neocortical slices with one particular specific HTR2A agonist (Mayer et al., 2019). More thorough studies using other agonists or serotonin are needed before reaching a final conclusion, especially due to the fact that multiple pathways are coupled to HTR2A receptor activation. In line with this, a recent study (Farrelly et al., 2019) identified a direct role of serotonin, which was independent from its function in neurotransmission and cellular signaling, in modifying histone proteins and, consequently, regulating gene expression. Findings on histone serotonylation have revealed a wide array of mechanisms for future investigations on cortical NPC proliferation modulated by serotonin (Farrelly et al., 2019).

## Outlook and Future Research Directions

Over the past few years, our view of NPCs during neocortical development has massively changed. The advancements in neuroimaging and single-cell transcriptomic analyses have enabled us to reveal more detailed profiling and characterization of different NPC types in different mammalian species (Fietz et al., 2012; Pollen et al., 2014; Florio et al., 2015; Nowakowski et al., 2017). This has provided foundations for further studies on the regulation of proliferation of different NPC types by neurotransmitters, and potentially in different model systems. This is true, in particular, when there are differential expression patterns of neurotransmitter receptors among different NPC populations or among different species, such as between mouse and human (Figure 1).

### Conditionally Gene-Modified Mouse Models

Various genetically engineered mouse models with disrupted neurotransmitter signaling have been generated to study the role of neurotransmitters in brain development (Ji et al., 1999; Di Giorgi-Gerevini et al., 2005; Côté et al., 2007; Cheng et al., 2010). However, systematically knocking out neurotransmitter-synthesizing or -degrading enzymes and neurotransmitter receptors in the whole organism is not ideal for studying the developing neocortex, since depleting or elevating the level of a particular neurotransmitter could potentially induce secondary alterations that may also bear significant impact on cortical development. Thus, it might be necessary to generate conditionally gene-modified mouse models that allow disruption of neurotransmitter signaling in a temporally and spatially more controlled manner. For example, glutamate decreases NPC proliferation through AMPA receptor activation, but increases NPC proliferation through mGluR5 activation (LoTurco et al., 1995; Di Giorgi-Gerevini et al., 2005). By conditionally knocking out the respective AMPA receptor and overexpressing mGluR5 exclusively in one specific NPC type, the proliferation-inhibiting AMPA receptor-coupled signaling could be abolished and the mGluR5-induced proliferation-stimulating signaling could be amplified, with the level of glutamate in the gene-modified animal remaining the same. These conditionally gene-modified mouse models could provide us with much more insight into the molecular mechanism of glutamate-regulated NPC proliferation and allow us to focus on studying the effects of glutamate in one particular NPC type.

### Human Brain Organoids as a Model System

Compared to human, rodents such as mouse and rat, the most commonly used experimental mammalian animals in developmental neuroscience, have a relatively small and smooth (lissencephalic) neocortex. In contrast, many primates, including human, have a folded (gyrencephalic) neocortex that is expanded in size (Florio and Huttner, 2014). Furthermore, the proportion of bRG among the BPs and their proliferative capacity are dramatically different between rodent and human (Fietz et al., 2010; Hansen et al., 2010). Therefore, in order to understand how human neocortex grows during development and expands during evolution, it is necessary to study the influence of neurotransmitters on NPC proliferation in fetal human neocortex. The development of brain organoids (Kadoshima et al., 2013; Lancaster et al., 2013) has opened up new avenues to study human neocortex development and evolution as well as neurodevelopmental disorders. Human brain organoids serve as a good, although not ideal, model which mimics certain aspects of the cytoarchitecture and cell-type composition of the developing human neocortex. Potential applications of wildtype and gene-modified brain organoids are feasible for studying the roles of neurotransmitters and their receptors in human NPC proliferation *ex vivo*.

### Neurotransmitters, Neocortex Malformations and Psychiatric Disorders

Neocortex malformations, which are thought to be caused by alterations of NPC proliferation and abundance, are featured in several neurological or psychiatric disorders such as epilepsy, Down syndrome and autism spectrum disorders (ASD) (Pinson et al., 2019). Some of these disorders also show altered levels of neurotransmitters in the CNS. For example, autism patients show a deceased level of GABA in the left perisylvian region of the auditory cortex (Rojas et al., 2014), and GABA receptor subunit genes on chromosome 15q11-q13 are considered risk factors for autistic disorders (Ashley-Koch et al., 2006). Moreover, altered levels of glutamate and serotonin as well as the functional deficiency or dysregulation of their receptors have also been suspected to be involved in ASD (Jamain et al., 2002; Yang et al., 2014; Zheng et al., 2016). It is plausible that the above mentioned neurotransmitter imbalance in ASD is caused by neocortex malformations with reduced abundance of neurotransmitter-secreting neurons, especially in cases linking maternal drug intake or exposure to developmental toxicants to neurodevelopmental disorders. For example, prenatal exposure to ethanol causes a spectrum of physical and mental dysfunctions in children, including pre- and postnatal growth delay, microcephaly, mental retardation and various behavioral abnormalities, which are due to the loss of specific cortical neurons and dysregulation of neuronal migration, such as GABAergic neurons (Shenoda, 2017), inhibition of the neurotrophic properties of glutamate, or the activation of specific GABA receptors (Ikonomidou et al., 2000).

One interesting question that can be raised from connecting neocortex malformations, induced by dysregulated NPC proliferation, with altered levels of neurotransmitters in developmental disorders is: Could disrupted neurotransmitter signaling during cortical development be the causative factor for disorders like autism? To further understand the influences of neurotransmitters in neurodevelopmental disorders like autism, we can now take advantage of the option to generate cerebral organoids from patient-derived induced pluripotent stem cells (iPSCs) to model the disorder and study the neocortex malformation and neurotransmitter imbalance involved in the disorder.

## Concluding Remarks

Over the past few years, the dissection of NPC cell biology during the development of the mammalian neocortex has given us substantial insights into the spatial and temporal control mechanisms of NPC proliferation by the concert of cell–intrinsic and cell–extrinsic factors. Studies on the developmental actions of neurotransmitters have also further advanced our understanding on how the growth of the neocortex can be affected by these extrinsic factors. Looking forward, with promising concepts and platforms being established, more comprehensive and integrative interpretations on how neurotransmitters maintain normal CNS development and protect against cortical dysfunction could be achieved. Learning more about the roles that neurotransmitters play during human cortical development will not only provide valuable knowledge for understanding our own cognitive abilities, but also shed light on the development of pharmacological interventions against a number of human neurodevelopmental disorders.

## Author Contributions

LX and WH wrote and edited the manuscript.

## Conflict of Interest

The authors declare that the research was conducted in the absence of any commercial or financial relationships that could be construed as a potential conflict of interest.
