# Corrigendum: Neurotransmitters as Modulators of Neural Progenitor Cell Proliferation During Mammalian Neocortex Development

**DOI:** 10.3389/fcell.2020.00515

**Published:** 2020-07-08

**Authors:** Lei Xing, Wieland B. Huttner

**Affiliations:** Max Planck Institute of Molecular Cell Biology and Genetics, Dresden, Germany

**Keywords:** neocortex, neurotransmitter, neural progenitor cell, cell proliferation, development

In the original article Albert et al. (2017) was not cited in the article. The citation has now been inserted in **Figure 1 legend** and should read:

Figure 1. Previously published sets of transcriptomic data (Florio et al., 2015; Albert et al., 2017) were analyzed here for the mRNA expression levels of neurotransmitter receptors in embryonic mouse and fetal human neocortex.

The authors apologize for this error and state that this does not change the scientific conclusions of the article in any way. The original article has been updated.
